# Gene-based analysis of angiogenesis, mitochondrial and insulin-related pathways in skeletal muscle of older individuals following nutraceutical supplementation

**DOI:** 10.1016/j.jff.2019.03.022

**Published:** 2019-05

**Authors:** Hannah Crossland, Suzette L. Pereira, Kenneth Smith, Bethan E. Phillips, Philip J. Atherton

**Affiliations:** a MRC-ARUK Centre for Musculoskeletal Ageing Research & NIHR Nottingham BRC, University of Nottingham, Royal Derby Hospital Centre, DE22 3DT, UK; b Abbott Nutrition Research and Development, Columbus, OH 43219, USA

**Keywords:** Skeletal muscle, Angiogenesis, Mitochondria, Gene expression

## Abstract

Cocoa flavanols and fish oil omega-3 fatty acids are two bio-active nutrients that may improve muscle microvascular function, insulin sensitivity and mitochondrial function in older adults. We assessed changes in gene expression of these pathways in muscle from two nutritional intervention studies in older healthy volunteers: (i) 6-weeks daily fish oil supplementation in older females (3.4 g/d; age: 64.4 ± 0.8 y, BMI: 26.2 ± 0.7 kg/m^2^), and (ii) 7-day daily cocoa flavanol supplementation in older males (1050 mg/d; age: 70.1 ± 0.9 y, BMI: 25.7 ± 0.6 kg/m^2^). There was a main effect of 6-weeks fish oil supplementation on angiogenesis gene expression, with no overall changes in mitochondrial or insulin signaling genes. 7-day cocoa supplementation elicited changes in extracellular matrix (ECM) related genes. Thus, the effects of fish oil supplementation on vascular remodeling in skeletal muscle, and ECM remodeling with cocoa supplementation have emerged as areas for future study.

## Highlights

• Fish oil supplementation in older females activated angiogenesis-related genes.
• Mitochondrial genes in muscle were not influenced by fish oil in older females.
• Cocoa supplementation in older males did not alter muscle angiogenesis genes.
• 7 day cocoa supplementation in older males caused changes in ECM genes in muscle.

## Graphical abstract

**Figure:**
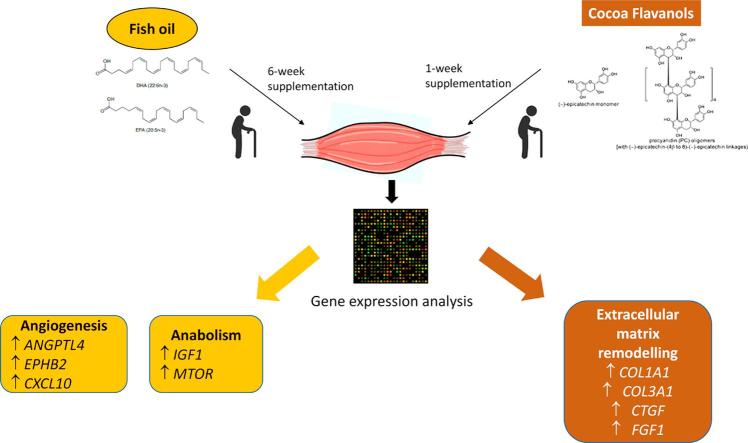

## 1 Introduction

Impaired skeletal muscle microvascular function, mitochondrial abundance/activity and insulin sensitivity are key features of ageing in humans, whereby age-related declines in muscle microvascular blood flow may contribute to impaired uptake of insulin/amino acids in response to feeding (Phillips et al., 2012). Flavonoid-rich foods such as green tea and cocoa, and fish oil derived omega-3 polyunsaturated fatty acids (PUFA) have emerged as potentially important bio-active nutrients that may protect against cardiovascular risk factors and metabolic disorders when dietary intake of these compounds is increased (Higginbotham and Taub, 2015, Siriwardhana et al., 2012).

In rats, supplementation with fish-derived fatty acids docosahexaenoic acid (DHA) and eicosapentaenoic acid (EPA) enhanced skeletal muscle blood flow during exercise (Stebbins, Hammel, Marshal, Spangenberg, & Musch, 2010). Similarly, in healthy young male humans, dietary fish oil supplementation for 4-weeks resulted in increased resting femoral arterial blood flow (Pearson, Johnson, & Robins, 2014). Fish oil supplements have also been implicated in positively influencing insulin action, and protecting against lipid-induced insulin resistance in human skeletal muscle (Stephens et al., 2014). In animal models, chronic fish oil supplementation has been demonstrated to increase whole-body endogenous glucose production (Kamolrat, Gray, & Carole Thivierge, 2013), and administration to mice fed a high-fat diet resulted in attenuation of insulin resistance through improvements in mitochondrial function (Martins et al., 2018). In older humans, long-term omega-3 PUFA treatment resulted in increased muscle mass and function (Smith et al., 2015), while the anabolic response to insulin and amino acid infusion was found to be enhanced with omega-3 PUFA administration in healthy young and middle-aged human volunteers (Smith et al., 2011).

Administration of dietary cocoa flavanols has also been linked with potential health benefits. Flavanols represent a group of polyphenolic plant-derived compounds that have been linked to improvements in cardiovascular health, primarily through lowering of blood pressure, stimulation of endothelial nitric oxide (NO) and alterations in mitochondrial function (Higginbotham & Taub, 2015). In a rat model of high-fat diet induced obesity (Gutiérrez-Salmeán et al., 2014), administration of (−)-epicatechin, the most abundant flavanol in cocoa, significantly improved cardiometabolic risk factors including weight gain and hypertriglyceridemia, and restored obesity-induced decreases in protein expression of several mitochondrial-associated factors, such as peroxisome proliferator-activated receptor coactivator alpha (PGC-1α), transcription factor A mitochondrial (TFAM) and uncoupling protein 1 (UCP1). Other studies, in both humans and rodents, have also demonstrated increases in markers of mitochondrial biogenesis following cocoa supplementation (Taub et al., 2012, Watanabe et al., 2014). Cocoa flavanols may also improve glucose tolerance and insulin sensitivity. In skeletal muscle cells, procyanidin-rich cocoa extracts enhanced glucose uptake and glycogen synthesis (Bowser et al., 2017), and in adult humans with hypertension, cocoa supplementation for 2-weeks enhanced insulin-stimulated brachial artery diameter, although there were no improvements in insulin resistance or blood pressure (Muniyappa et al., 2008). In older humans, acute treatment with cocoa flavanols improved both micro- and macro-vascular responses to nutrition, but did not improve protein synthetic responses to feeding (Phillips et al., 2016).

Thus, despite growing evidence supporting a role for both fish oil and cocoa flavanol supplements in impacting cardiovascular and metabolic health in humans, the mechanisms by which they mediate these effects remain unclear, particularly in ageing. Therefore, the aim of the current study was to evaluate the effect of 1-week cocoa flavanol supplementation, and chronic fish oil supplementation, on gene expression responses related to angiogenesis, mitochondrial function and insulin signaling pathways in the skeletal muscle of older adults. We hypothesized that fish oil and cocoa flavanol supplementation would influence the expression of genes important in regulating all of these processes.

## 2 Material and methods

### 2.1 Nutritional intervention studies in older adults: (i) fish oil study

Gene expression analysis was carried out on skeletal muscle from two human clinical studies (*n* = 8 per group): in study 1 (NIH Clinical Trials Identifier (NCT): 02505438), skeletal muscle biopsies (from the *m. vastus lateralis* mid-belly*)* were collected in a rested, post-absorptive state before and after 2- and 6-weeks fish oil supplementation (3.4 g/d; 90% EPA; Minami Nutrition PLUSEPA®, Belgium; 4–6 h after the final dose of fish oil) from older females (age: 64.4 ± 0.8 y, BMI: 26.2 ± 0.7 kg/m^2^). Samples were washed in ice-cold saline, before being snap frozen in liquid nitrogen and stored at −80 °C until further analysis. Subjects were instructed to consume the daily dose in the morning with food. The dose was chosen based upon previous publications demonstrating improvements in muscle mass and function and feeding response of muscle protein synthesis (Smith et al., 2011, Smith et al., 2015). All subjects were aged 65-75y. Subjects were all pre-screened, with exclusions for BMI > 30, a history or symptoms of cardiovascular, respiratory or metabolic disorders or those having taken medications known to affect muscle metabolism (e.g. NSAIDS, statins, etc.) chronically. All subjects gave their written, informed consent to participate after all procedures and risks were explained. Each study was approved by The University of Nottingham Faculty of Medicine and Health Sciences Research Ethics Committee and complied with the Declaration of Helsinki.

### 2.2 Nutritional intervention studies in older adults: (ii) cocoa study

In study 2 (NCT: 01734616), post-absorptive *m. vastus lateralis* biopsies were taken from a group of older males (age: 70.1 ± 0.9 y, BMI: 25.7 ± 0.67 kg/m^2^) who received 350 mg cocoa flavanols (CocoaVia®, Mars Inc, USA) 3 times each day for 7 days, compared to a control (no supplementation) group. Subjects were instructed to take the supplement with breakfast, lunch and dinner each day to try and ensure some degree of standardisation across subjects. The 350-mg 3x/d dose was chosen as this dose is similar to the acute dose at which improvements in indices of cardiovascular function have been observed (Mogollon et al., 2013, Sansone et al., 2015) and closely represents the dose given during chronic intervention studies (Balzer et al., 2008, Engler et al., 2004, Muniyappa et al., 2008). Samples were washed in ice-cold saline, before being snap frozen in liquid nitrogen and stored at −80 °C until further analysis. In relation to the final dose of the supplement, biopsies were taken ∼24 h after the last dose.

All subjects were aged 65–75y. All subjects were initially screened by means of a medical questionnaire, physical examination, and resting electrocardiography, with exclusions for overt muscle wasting (>2 SD below age norms) (Baumgartner et al., 1998), metabolic, respiratory, or cardiovascular disorders or other signs and symptoms of ill-health. All subjects had normal blood chemistry, were normotensive (blood pressure < 140/90), and were not prescribed medication. All subjects performed activities of daily living and recreation but did not routinely participate in formal exercise regimes.

### 2.3 RNA extraction and cDNA synthesis

Tissue homogenization for RNA extraction was performed using 10–15 mg of muscle tissue in TRIzol™ (Invitrogen, Thermo Fisher Scientific) using a Tissue Lyser II (Qiagen). RNA extraction was performed using the manufacturer’s protocol, and RNA quantity and quality was assessed using a NanoDrop 2000 (Thermo Fisher Scientific). The high capacity cDNA synthesis kit (Applied Biosystems, Thermo Fisher Scientific) was used to reverse transcribe RNA samples (500 ng), which were then diluted 1:5.

### 2.4 Taqman™ array panel

For evaluation of angiogenesis-related genes, cDNA samples mixed with TaqMan Universal Master Mix II (Applied Biosystems, Thermo Fisher Scientific) were loaded onto pre-designed 384-well microfluidic cards; the TaqMan™ Array human angiogenesis panel (4378710, Applied Biosystems, Thermo Fisher Scientific). Plates were run on a ViiA™ 7 real-time PCR system (Thermo Fisher Scientific). The array contained 94 assays and one housekeeping gene (*GAPDH*). Any genes with amplification of later than 34 Ct were not analyzed.

### 2.5 Targeted real-time PCR analysis

Supplementary data associated with this article can be found, in the online version, at https://doi.org/10.1016/j.jff.2019.03.022.

For targeted real-time PCR analysis, 1 µl of cDNA (in duplicate) was added into 384 well plates with gene specific primers (see Supplementary Table 1) and SYBR Select Master Mix (Thermo Fisher Scientific) in a total volume of 7 µl. The ΔΔCt method (Schmittgen & Livak, 2008) was used to quantify mRNA expression, with *GAPDH* being used for normalization.

**Supplementary Table 1:** 

### 2.6 Statistical analyses

Genes were sub-categorized based on ontology and 2-way ANOVA used to assess main effects of each treatment. Sidak’s tests (correcting for multiple comparisons) were used to determine any individual changes in gene expression between groups within each study.

## 3 Results

### 3.1 Changes in mitochondrial and insulin signaling gene expression

**Fig. 1:**
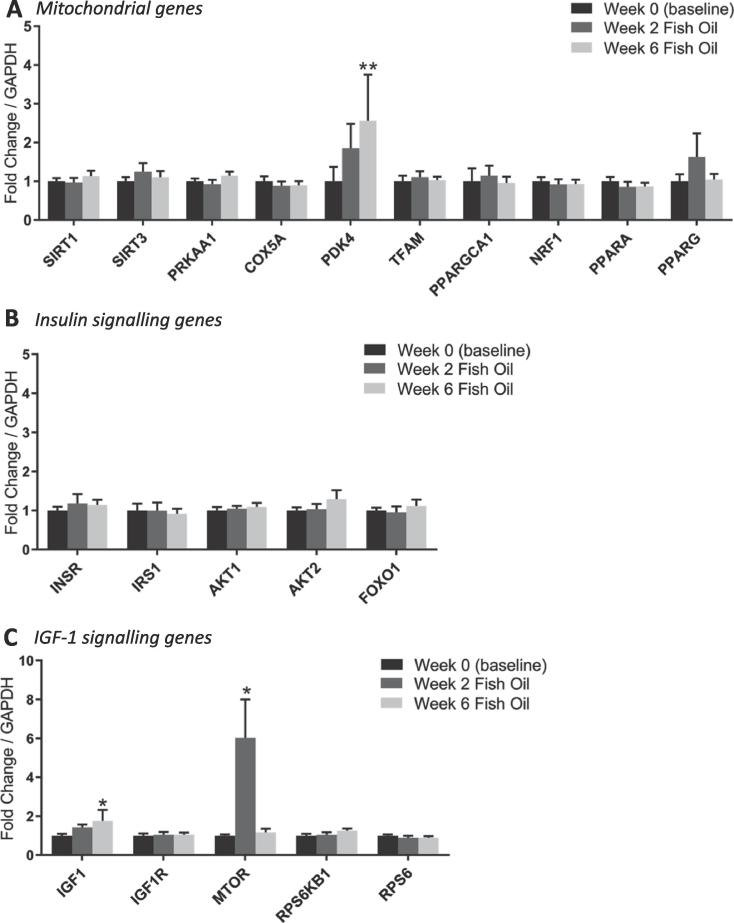
Expression of mitochondrial (A), insulin (B) and IGF-1 (C) signaling-related genes in skeletal muscle of older females before and after 2- and 6-weeks fish oil supplementation. Data are normalized to GAPDH expression and expressed as mean ± SEM (n = 8 per group). *; *P* < 0.05, **; *P* < 0.01 vs. week 0 (baseline).

Analysis of mitochondrial and insulin/insulin-like growth factor (IGF)-1 signaling genes in the skeletal muscle of older females after 6-weeks fish oil supplementation showed no main effects, however there were significant increases in pyruvate dehydrogenase kinase 4 (*PDK4*) (2.6 ± 1.1-fold; *P* < 0.01 vs. baseline) and *IGF1* (1.8 ± 0.6-fold; *P* < 0.05 vs. baseline) gene expression (Fig. 1). Based on these findings, in order to explore the temporal nature of these changes, muscle biopsies that were collected after 2-weeks of fish oil supplementation were also analyzed, however there were no overall effects on either mitochondrial or insulin signaling associated genes in this time-frame, although there was an increase in mechanistic target of rapamycin (*MTOR*) expression (6.0 ± 1.9-fold; *P* < 0.05 vs. baseline; Fig. 1).

**Fig. 2:**
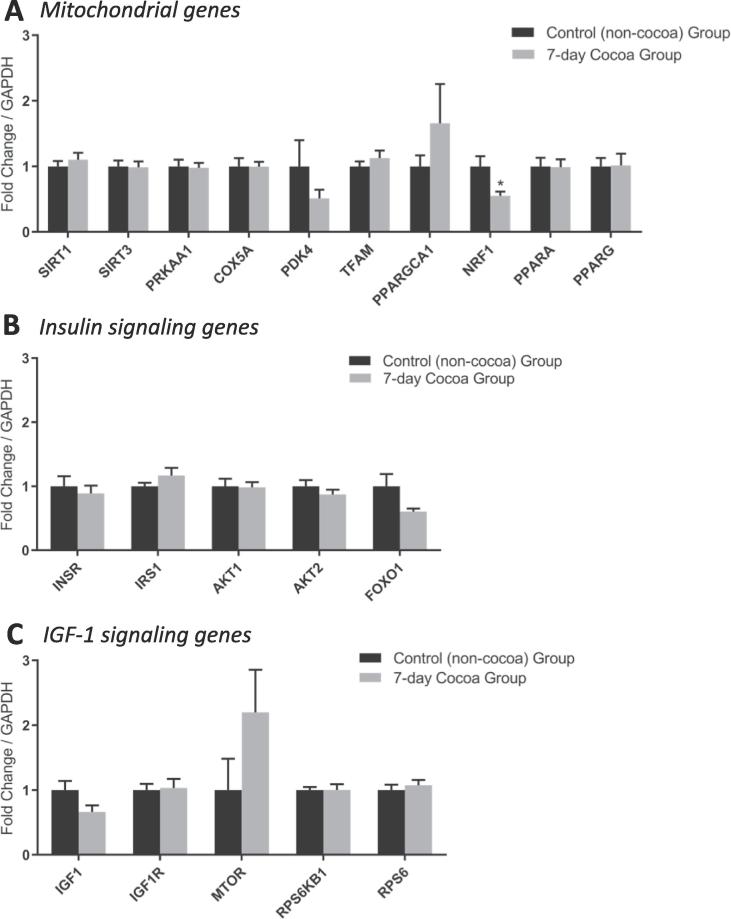
Expression of mitochondrial (A), insulin (B) and IGF-1 (C) signaling-related genes in skeletal muscle of older males, following 7-days cocoa flavanol supplementation. Data are normalized to GAPDH expression and expressed as fold change versus basal non-cocoa group (mean ± SEM; n = 8 per group). *; *P* < 0.05 vs. control (non-cocoa) group.

Analysis of mitochondrial, insulin signaling and IGF-1 signaling pathways in the skeletal muscle of older males after 7-days of cocoa flavanol supplementation showed no overall difference in mitochondrial or insulin/IGF-1-related gene expression compared to a no supplementation control group, although *NRF1* expression was significantly lower (0.55 ± 0.05-fold; *P* < 0.05 vs. non-cocoa; Fig. 2).

### 3.2 Changes in angiogenesis gene expression

**Fig. 3:**
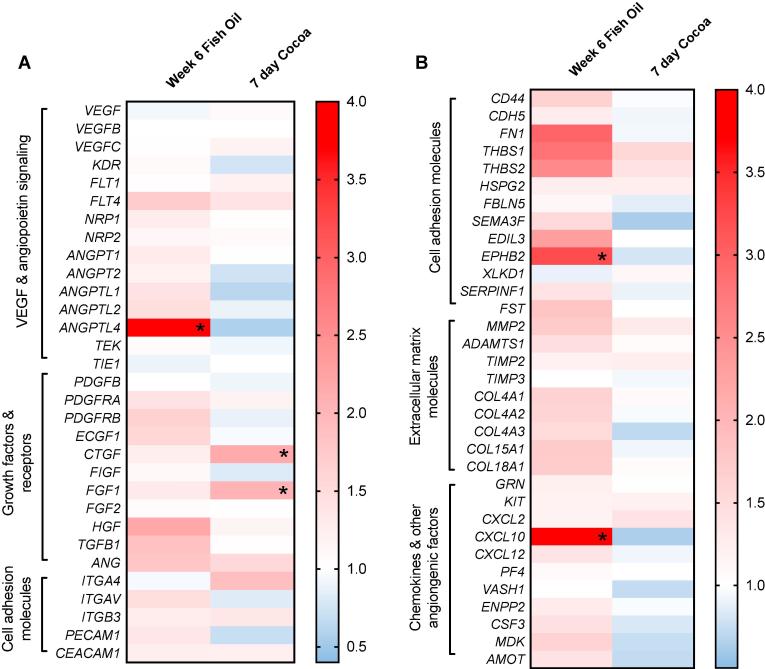
Heat map representing expression of angiogenesis-related genes in skeletal muscle of older females before and after 6-weeks fish oil supplementation, and in older males following 7-days cocoa flavanol supplementation. Data are normalized to GAPDH expression and expressed as mean fold change ± SEM (n = 8 per group). Colors represent magnitude of change in expression of each gene (red = up-regulated; blue = down-regulated). *; *P* < 0.05, vs. week 0 (baseline) for fish oil study and vs. control (non-cocoa) group for cocoa study. There was a main effect of fish oil (*P* < 0.05 by ANOVA) on overall gene changes.

Fish oil supplementation for 6-weeks in older females resulted in a main effect on angiogenesis gene expression in skeletal muscle (Fig. 3). Individual gene analysis revealed significant increases in Angiopoeitin-like 4 (*ANGPTL4*) (3.7 ± 1.8-fold; *P* < 0.01 vs. baseline), Ephrin type-B receptor 2 (*EPHB2*) (3.2 ± 1.1-fold; *P* < 0.05 vs. baseline) and C-X-C Motif Chemokine Ligand 10 (*CXCL10*) (3.8 ± 1.9-fold; *P* < 0.01 vs. baseline).

Conversely, 1-week cocoa flavanol supplementation, which did increase leg blood flow (femoral artery via Doppler Ultrasound) responses to nutrition (cocoa: 0.48 ± 0.04 vs. 0.52 ± 0.04 l/min, non-cocoa: 0.32 ± 0.04 vs. 0.32 ± 0.04 l/min), did not demonstrate a main effect on angiogenesis gene expression in skeletal muscle (Fig. 3), however expression of *CTGF* (2.1 ± 0.4-fold) and *FGF1* (2.0 ± 0.4-fold) was significantly higher in the cocoa group (both *P* < 0.01 vs. non-cocoa).

### 3.3 Changes in extracellular matrix (ECM) related gene expression

**Fig. 4:**
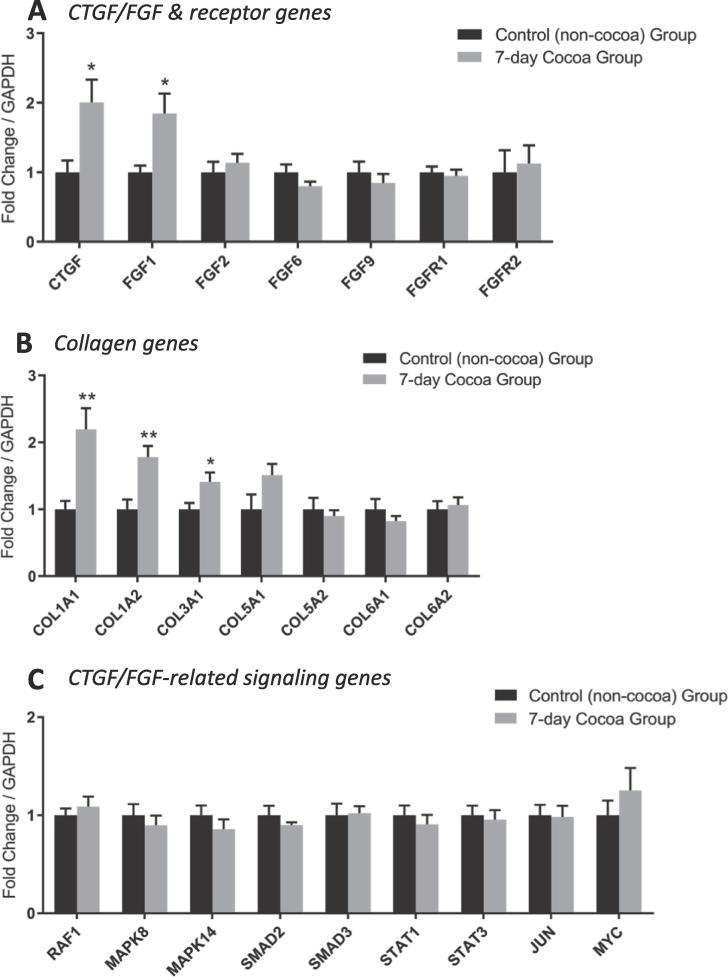
Expression of CTGF and FGF-related genes (A), collagen-related genes (B) and FGF signaling-related genes (C) in skeletal muscle of older males, following 7-days cocoa flavanol supplementation. Data are normalized to GAPDH expression and expressed as fold change versus control (non-cocoa) group (mean ± SEM; n = 8 per group). *; *P* < 0.05, **; *P* < 0.01 vs. non-cocoa group.

As a follow-on to the observed changes in angiogenesis-related gene expression with cocoa supplementation, analysis into pathways associated with connective tissue growth factor (CTGF) and fibroblast growth factor (FGF) signaling showed that 7-day cocoa treatment in older males was associated with heightened *CTGF* expression in skeletal muscle (thus confirming the gene card data; Fig. 4), as well as heightened *FGF1* (1.8 ± 0.4-fold; *P* < 0.05 vs. non-cocoa), *COL1A1* (2.2 ± 0.4-fold; *P* < 0.01 vs. non-cocoa), *COL1A2* (1.8 ± 0.2-fold; *P* < 0.01 vs. non-cocoa) and *COL3A1* (1.4 ± 0.1-fold; *P* < 0.05 vs. non-cocoa) expression (Fig. 4).

## 4 Discussion

Dietary supplementation with both cocoa flavanols and omega-3 PUFA have consistently been associated with numerous health benefits in humans, including improvements in cardiovascular function, and glucose and lipid metabolism (Gutiérrez-Salmeán et al., 2014, Higginbotham and Taub, 2015, Martins et al., 2018, Siriwardhana et al., 2012). Since ageing is associated with declines in muscle microvascular function, insulin sensitivity and mitochondrial function, it follows that dietary supplementation with either fish oil or cocoa flavanols could exert beneficial effects in the skeletal muscle of older individuals. In the present study, we aimed to examine whether fish oil or cocoa flavanol supplementation positively influenced expression of genes related to angiogenesis, mitochondrial biogenesis/function and IGF-1/insulin signaling pathways in the skeletal muscle of older adults.

### 4.1 Changes in angiogenesis, mitochondrial and insulin signaling gene expression in skeletal muscle following fish oil supplementation in older adults

Consistent with previous evidence that dietary fish oil can have beneficial effects related to improving muscle blood flow (Pearson et al., 2014), 6-weeks supplementation in older females caused a significant change in genes important for the regulation of angiogenesis in muscle, with individual significant changes in *ANGPTL4*, *EPHB2* and *CXCL10*. *ANGPTL4* has been implicated in functions related to regulation of angiogenesis, as well as glucose homeostasis and lipid metabolism (Xu et al., 2005), while *EPHB2* encodes a transmembrane glycoprotein that is implicated in diverse cellular processes including cell migration, cell-cell communication and vascular development (Kullander & Klein, 2002). *CXCL10* is thought to act as a chemoattractant, an important feature in angiogenesis regulation (Strieter, Burdick, Gomperts, Belperio, & Keane, 2005).

Contrary to our original hypothesis, there was no effect of 6-weeks fish oil supplementation on gene expression related to mitochondrial function/abundance or insulin signaling, with temporal analysis at an earlier time-point (2-weeks) leading to the same conclusion. This is in line with results from previously reported longer-term (12-weeks) fish oil supplementation in young adults which did not find increase in muscle maximal mitochondrial respiratory function or levels of the electron transport chain proteins, but did observe increased mitochondrial sensitivity to ADP (Herbst et al., 2014). It is possible that despite our findings, molecular changes may have occurred earlier than the 2-week time point. Measures of mitochondrial proteins and/or mitochondrial enzyme activity would be required to confirm whether chronic fish oil supplementation can induce a functional change in the mitochondrial abundance and/or activity in the skeletal muscles of older females. Gene analysis did, however, reveal that fish oil supplementation increased gene expression of *IGF1* (at 6-weeks) and *MTOR* (at 2-weeks), potentially indicating an anabolic effect in skeletal muscle, which is consistent with previous studies (Smith et al., 2011, Smith et al., 2015). It should be acknowledged that while biopsies were taken from the rested leg, the participants in the original study also performed 6-week unilateral resistance exercise training, therefore we cannot rule out exercise potentially influencing the current gene expression data. Nevertheless, the beneficial effects of fish oil supplementation in older adults may be, in part, related to their effects on muscle angiogenesis regulation and anabolism.

### 4.2 Changes in angiogenesis, mitochondrial and insulin signaling gene expression in skeletal muscle following cocoa flavanol supplementation

In humans, cocoa flavanols have been consistently shown to exert beneficial effects related to vasodilation and blood flow (Grassi et al., 2015), and we previously demonstrated in older adults that acute treatment with cocoa flavanols improved micro- and macro-vascular responses to nutrition (Phillips et al., 2016). We hypothesized therefore that in older adults, dietary cocoa supplementation would exert beneficial effects related to muscle angiogenesis-related pathways.

We did not assess whether 7-day cocoa supplementation caused an increase in muscle capillary growth, but our findings suggest that molecular responses occurred early, as 7-days cocoa supplementation showed no differences in angiogenesis-related genes compared to our control group. There was however, higher *CTGF* and *FGF1* mRNA expression with 1-week cocoa treatment in older males relative to control, leading us to further evaluate these pathways in the cocoa supplementation group. *CTGF* encodes an ECM-associated growth factor that, through its interactions with a number of integrins, cell surface heparan sulfate proteolgycans (HPSGs) and growth factors, has many important roles related to cell adhesion, angiogenesis, wound healing and tissue remodeling (Brigstock, 2002, Lipson et al., 2012). Similarly, the FGF pathway has functions in multiple cell types related to modulation of angiogenesis, cell growth and tissue repair (Basilico and Moscatelli, 1992, Beenken and Mohammadi, 2009). FGF proteins are associated with multiple downstream signaling pathways, including Ras/mitogen-activating protein kinase (MAPK), PI3K/AKT and phospholipase C gamma (PLCγ) (Ornitz & Itoh, 2015).

We found that key collagen coding genes (*COL1A1*, *COL1A2* and *COL3A1*) showed heightened expression in muscle following 7-day cocoa flavanol supplementation, consistent with an ECM remodeling response. It was not possible to determine whether these changes were directly required for and/or related to new capillary formation, but these findings clearly point towards changes in cell adhesion, angiogenesis and ECM deposition/remodeling being key features of the physiological effects of cocoa flavanol supplementation in ageing skeletal muscle. There is currently little known about the potential role of polyphenols in ECM remodeling, although a previous study found that different flavonoids showed differential effects on collagen synthesis in human fibroblasts (Stipcevic, Piljac, & Berghe, 2006), and some studies have linked flavonoids with inhibition of ECM protein production (Vinayagam & Xu, 2015). Thus, cocoa polyphenols may exert important tissue remodeling effects on skeletal muscle in older adults, although more work is required to understand the precise effects that polyphenols have on the skeletal muscle of older humans. A recent study (Barrera-Reyes et al., 2018) demonstrated that cocoa intake in healthy humans was associated with gene expression changes in peripheral mononuclear cells (PBMCs) related to regulation of ROS production, calcium handling and inflammatory responses. Thus, wider exploration of the pathways regulated by flavanols in older skeletal muscle would provide more insight into their potential health benefits in ageing.

In high-fat diet fed mice, procyanidin extract caused a suppression of hyperglycemia and fat accumulation in adipose tissue (Yamashita, Okabe, Natsume, & Ashida, 2012), while in rats bred for innate low running capacity, chronic treatment with (−)-epicatechin resulted in increased muscle capillarity as well as increased protein expression of mitochondrial biogenesis proteins (PGC-1α and TFAM) (Hüttemann et al., 2013). In human studies, long-term cocoa supplementation has been shown to enhance markers of mitochondrial content and function in skeletal muscle (Taub et al., 2012, Taub et al., 2016). Our analyses revealed that 7-day supplementation with cocoa flavanols did not result in changes in gene expression associated with mitochondrial biogenesis or function. This is somewhat in contrast to previously published studies (Taub et al., 2012) and could be due the shorter duration of our intervention (7-day treatment) versus a longer time-frame (e.g. 3 months (Taub et al., 2012)).

### 4.3 Conclusions

Our data indicates that following chronic (6-week) fish oil supplementation in older females, we did not observe any changes in genes important for mitochondrial or insulin signaling related pathways but did observe an overall change in angiogenesis-related gene expression. Furthermore, increases in key anabolic related genes with fish oil administration are consistent with previous observations (Smith et al., 2011). While previous work has indicated that fish oil has a greater effect in females (Da Boit et al., 2017), we acknowledge the limitations in the present study in only studying females. Similarly, while cocoa studies have shown that both males and females achieve beneficial effects (Sansone et al., 2015), but also perhaps more so in men (Ostertag et al., 2013), more studies are required in both genders. Like in the fish oil study, with 7-day cocoa supplementation we were unable to detect changes in insulin or mitochondrial gene expression, although it should be noted that differences between the cocoa and fish oil studies (i.e. in terms of supplementation timings/doses, difference in gender and study design), could all have played a role in impacting the present gene expression results. Of interest is the observation that a cocoa supplementation intervention resulted in ECM remodeling within aged muscle which indicates structural changes starting to occur within just 7 days of intervention. Whether these structural changes are tied to increased capillarization within an aged muscle remains to be evaluated in future long-term cocoa intervention studies.

## Ethical statements

All subjects gave their written, informed consent to participate after all procedures and risks were explained. Each study was approved by The University of Nottingham Faculty of Medicine and Health Sciences Research Ethics Committee and complied with the Declaration of Helsinki.
