# Supplementary material for: Gene-based analysis of angiogenesis, mitochondrial and insulin-related pathways in skeletal muscle of older individuals following nutraceutical supplementation
Source: J Funct Foods. 2019 May;56:216–23. doi: 10.1016/j.jff.2019.03.022 (PMC6559337; doi:10.1016/j.jff.2019.03.022)
Supplement: Supplementary file 1 [file mmc1.docx]

**Supplementary Table 1**

| **Gene Symbol** | **Gene Name** | **Forward Primer** | **Reverse Primer** |
| --- | --- | --- | --- |
| *SIRT1* | Sirtuin 1 | AGGCCACGGATAGGTCCATA | GTGGAGGTATTGTTTCCGGC |
| *SIRT3* | Sirtuin 3 | GACATTCGGGCTGACGTGAT | CAATGTCGGGCTTCACAACG |
| *PRKAA1* | Protein Kinase AMP-Activated Catalytic Subunit Alpha 1 | TGGAAGGCTGGATGAAAAAGA | GGACCACCATATGCCTGTGA |
| *COX5A* | Cytochrome C Oxidase Subunit 5A | TGGCTATCCAGTCAGTTCGC | TGTTACCCAGCGAGCATCAA |
| *PDK4* | Pyruvate Dehydrogenase Kinase 4 | ACTCGGATGCTGATGAACCA | TCAAAGGCATCTTGGACCAC |
| *TFAM* | Transcription Factor A, Mitochondrial | CCAAAAAGACCTCGTTCAGCTT | CTTCAGCTTTTCCTGCGGTG |
| *PPARGCA1* | PPARG Coactivator 1 Alpha | GACACCCTCTTCTCTTCCTTCTTT | GCAGTCCAGGGGCAGAAAAGT |
| *NRF1* | Nuclear Respiratory Factor 1 | CAGCCGCTCTGAGAACTTCA | CGGTGTAAGTAGCCACATGGA |
| *PPARA* | Peroxisome Proliferator Activated Receptor Alpha | GCTGGTGTATGACAAGTGCG | CGAATCGCGTTGTGTGACAT |
| *PPARG* | Peroxisome Proliferator Activated Receptor Gamma | CGTGGCCGCAGATTTGAAAG | TCGTTAAAGGCTGACTCTCGT |
| *INSR* | Insulin Receptor | TCTACGTGACAGACTATTTAGACG | CCATCTGGCTGCCTCTTTCT |
| *IRS1* | Insulin Receptor Substrate 1 | ACATCACAGCAGAATGAAGACCT | TGAAATGGATGCATCGTACC |
| *AKT1* | AKT Serine/Threonine Kinase 1 | GAGAAGAAGCTCAGCCCACC | TCCACACACTCCATGCTGTC |
| *AKT2* | AKT Serine/Threonine Kinase 2 | TTGCCAAGGATGAAGTCGCT | CGTGGGTCTGGAAGGCATAC |
| *FOXO1* | Forkhead Box O1 | CTTTGACAATGTGTTGCCCA | GTGTAACCTGCTCACTAACCCT |
| *IGF1* | Insulin Like Growth Factor 1 | AAATCAGCAGTCTTCCAACCC | GTGTGCATCTTCACCTTCAAGAAA |
| *IGF1R* | Insulin Like Growth Factor 1 Receptor | TGTCCAGGCCAAAACAGGAT | CAACCCTCCCACGATCAACA |
| *MTOR* | Mechanistic Target Of Rapamycin Kinase | AAGCCGCGCGAACCTC | TGGTTTCCTCATTCCGGCTC |
| *RPS6KB1* | Ribosomal Protein S6 Kinase B1 | CCATGAAGGTGCTTAAAAAGGCAA | TTCCACCAGTCTGAAAGGCA |
| *RPS6* | Ribosomal Protein S6 | TGGGTGAAGAATGGAAGGGTTA | CCTGCTTCATGGGGAAACCT |
| *CTGF* | Connective Tissue Growth Factor | ACCAATGACAACGCCTCCTG | TGCCCTTCTTAATGTTCTCTTCC |
| *FGF1* | Fibroblast Growth Factor 1 | CGGCTCACAGACACCAAATG | TCCCATTCTTCTTGAGGCCA |
| *FGF2* | Fibroblast Growth Factor 2 | GCTGTACTGCAAAAACGGGG | TAGCTTGATGTGAGGGTCGC |
| *FGF6* | Fibroblast Growth Factor 6 | AGATTGTACGCAACGCCCAG | GGCAATGTAGGTCCCTTGGT |
| *FGF9* | Fibroblast Growth Factor 9 | GGGGAGCTGTATGGATCAGAAA | GTATCGCCTTCCAGTGTCCA |
| *FGFR1* | Fibroblast Growth Factor Receptor 1 | AAACCGTATGCCCGTAGCTC | GAACTTCACTGTCTTGGCAGC |
| *FGFR2* | Fibroblast Growth Factor Receptor 2 | CACGACCAAGAAGCCAGACT | TGGACTCAGCCGAAACTGTTA |
| *COL1A1* | Collagen Type I Alpha 1 Chain | ACTGGTGAGACCTGCGTGTA | GCCGCCATACTCGAACTGGA |
| *COL1A2* | Collagen Type I Alpha 2 Chain | CAGCCGGAGATAGAGGACCA | CAGCAAAGTTCCCACCGAGA |
| *COL3A1* | Collagen Type III Alpha 1 Chain | TGGAGGATGGTTGCACGAAA | ACAGCCTTGCGTGTTCGATA |
| *COL5A1* | Collagen Type V Alpha 1 Chain | CTGTGCTACCAAGAAAGGCTA | CTAGCCCATGAAGCAAGC |
| *COL5A2* | Collagen Type V Alpha 2 Chain | ACATGATGGCAAACTGGGCG | TTCACCATATCCTTCATCCTCGT |
| *COL6A1* | Collagen Type VI Alpha 1 Chain | TAAAGGCTACCGAGGCGATG | GCCGTCTTCTCCCCTTTCAC |
| *COL6A2* | Collagen Type VI Alpha 2 Chain | CCTCGGGACCAGGACTTCAG | GGTAGTGTCCGGCGAGATG |
| *RAF1* | Raf-1 Proto-Oncogene, Serine/Threonine Kinase | GGATGATTGAGGATGCAATTCG | TTGGGCTCAGATTGTTGGGG |
| *MAPK8* | Mitogen-Activated Protein Kinase 8 | TGGACTTGGAGGAGAGAACCA | ACTGCTGCACCTGTGCTAAA |
| *MAPK14* | Mitogen-Activated Protein Kinase 14 | CCCGCTTATCTCATTAACAGGATG | CAAGTCGACAGCCAGGGGA |
| *SMAD2* | SMAD Family Member 2 | TGTGTTACCATACCAAGCACT | GGCCTGTTGTATCCCACTGA |
| *SMAD3* | SMAD Family Member 3 | AGCTGTGTGAGTTCGCCTTC | ACACAGGAGGTAGAACTGGTG |
| *STAT1* | Signal Transducer And Activator Of Transcription 1 | CTGTGCGTAGCTGCTCCTTT | CACTGAGACATCCTGCCACC |
| *STAT3* | Signal Transducer And Activator Of Transcription 3 | GAAACAGTTGGGACCCCTGA | AGGTACCGTGTGTCAAGCTG |
| *JUN* | Jun Proto-Oncogene, AP-1 Transcription Factor Subunit | GCGCCTGATAATCCAGTCCA | CCCTCCTGCTCATCTGTCAC |
| *MYC* | MYC Proto-Oncogene, BHLH Transcription Factor | GGTAGTGGAAAACCAGCAGCC | TCTCCTCCTCGTCGCAGTA |
